# Identification of beta cell dysfunction at the pre-symptomatic stage of diabetes mellitus by novel analytical system: liquid biopsy measurements in femtograms

**DOI:** 10.1007/s13167-017-0079-5

**Published:** 2017-02-13

**Authors:** Kurt Krapfenbauer

**Affiliations:** Department of Cranio-Maxillofacial and Oral Surgery, Medical University of Vienna, Waehringerguertel 18-20, A-1090 Vienna, Austria

**Keywords:** Biochip, Clinical applications, Miniaturization, Multiplexing, Personalized and predictive medicine, Novel detection conjugate, Diabetes mellitus, Pancreatic polypeptide, Beta cell failure

## Abstract

**Background:**

Diabetes mellitus is produced and progresses as a consequence of complex and gradual processes, in which a variety of alterations of the endocrine pancreas, are involved and which mainly result in beta cell failure. Those molecular alterations can be found in the bloodstream, which suggests that we could quantify specific biomarkers in plasma or serum by very sensitive methods before the onset diabetes mellitus is diagnosed. However, classical methods of protein analysis such as electrophoresis, Western blot, ELISA, and liquid chromatography are generally time-consuming, lab-intensive, and not sensitive enough to detect such alteration in a pre-symptomatic state of the disease.

**Method:**

A very sensitive and novel analytical detection conjugate system by using the combination of polyfluorophor technology with protein microchip method was developed.

**Results:**

This innovative system facilitates the use of a very sensitive microchip assays that measure selected biomarkers in a small sample volume (10 μL) with a much higher sensitivity (92%) compare to common immune assay systems. Further advances of the application of this technology combine the power of miniaturization and faster quantification (around 10 min).

**Conclusion:**

The power of this technology offers great promise for point-of-care clinical testing and monitoring of specific biomarkers for diabetes in femtogram level in serum or plasma. In conclusion, the results indicate that the technical performance of this new technology is valid and that the assay is able to quantified PPY-specific antigens in plasma at femtogram levels which can be used for identification of beta cell dysfunction at the pre-symptomatic stage of diabetes mellitus.

## Introduction

According to the World Health Organization, diabetes diseases will increase by 45% from 2007 to 2030 due to the demographic increase and population aging [1]. For that reason, many efforts have been made to find sensitive and specific biomarkers for early diagnosis, prognosis, and management of pre-diabetes patients during treatment and follow-up.

The presence of small amounts of circulating specific biomarkers in plasma and serum is not a new finding. The verification that such amounts are significantly increased in diabetes patients, and that biomarkers might carry a variety of alterations related to diabetes development and progression, has aroused great interest in the scientific community in the last decades. Such alterations potentially reflect changes that occur during the onset of pancreatic beta cell dysfunction, and include secretion of small peptides, or post translation of proteins that can be used as specific related biomarkers. These findings have led to many efforts toward the implementation of new clinical immune assays. However, the quantities of these biomarkers in plasma are very low meaning that we need very high sensitive and specific analytical systems. In the present article, we describe the main findings related to the development of a novel immune assays by using the so-called polyfluorophor technology which can be used for early diagnosis, prognosis, and monitoring of diabetes mellitus, most of which appear promising. However, due to the lack of harmonization of laboratory techniques, the heterogeneity of disease progression and the small number of recruited patients in most of those studies, there has been a poor translation of basic research into clinical practice [2, 3]. In addition, many aspects remain unknown, such as the release mechanisms of secretion of specific beta cell peptides, their biological function, and the way by which they circulate in the bloodstream. It is therefore expected that in the coming years, an improved understanding of the relationship between circulation biomarkers and the molecular biology of diabetes will lead to better diagnosis, management, and treatment.

## Customer need for the identification of beta cell failure

**Fig. 1:**
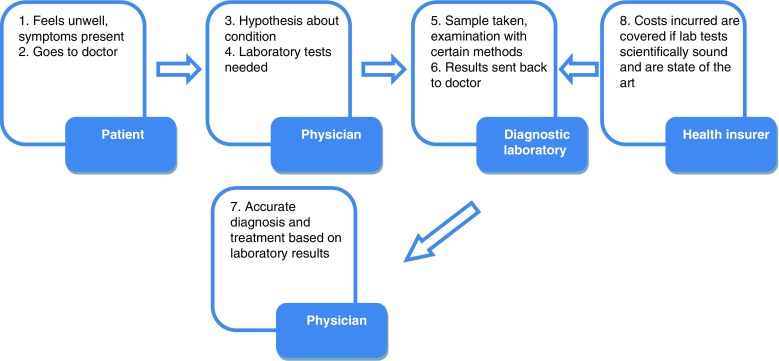
Traditional process of diagnosis

The traditional process of diagnosing a disease is a complicated one and involves up to four different parties, depending on the condition (Fig. 1).

A patient will consult his physician with certain symptoms already present. Based on these symptoms, a physician will make a hypothesis what the disease may be. However, to prove his theory, he often requires specialized laboratory tests for example to test the beta cell function by using an oral glucose tolerant test (OGTT). So, several time-depending blood samples during the OGTT will be taken and examined by any diagnostic laboratory. When the test results are available, a treatment will be assigned by the physician. This treatment may prove to be accurate and leads to a stabilization process or may be inaccurate and will require further investigation. On the monetary side, as far as the patient is insured, the costs for diagnosis are born by the health insurer, i.e., patients have to pay indirectly for diagnostic tests through their health insurance contributions. However, the health insurer is only willing to pay if laboratories are using state-of-the-art diagnostic methods.

There are several problems arising from this traditional method of diagnosing for the parties involved, which will be considered as paying and receiving customers.

### Limitation of the current diagnostic systems

#### Diagnostic laboratories

- Current diagnostic methods are time consuming. They normally require hours until the result can be validated. Furthermore, high costs can be associated with diagnostic instruments (especially purchasing costs). Therefore, it is clear that a faster and cost-efficient instrument is needed. This would cut processing times and associated costs.
- Current diagnostics instruments are not always able to deliver an accurate diagnosis to the doctor (e.g., beta cell failure associated with diabetes must be developed enough in order to be identified by an OGTT) and may lead to a wrong treatment. So, an accurate diagnostic instrument is also needed in those institutions.

#### Public and private health insurers

The late application of current diagnostic methods after symptoms already have occurred can imply higher treatment costs (longer hospital stays etc.) especially if diseases are diagnosed in a late stage [4]. As a result, earlier and accurate treatment would significantly shorten those costs. Many public health insurers are currently experiencing financial deficits and cannot sustain their operations in the long run without cash injections. Thus, such an early diagnostic instrument may solve this problem.

### Receiving customers

#### Patients

- Current diagnostic methods are used when symptoms are already present. Therefore, diseases are often diagnosed in a late stage, which means a lower healing chance for patients. The sooner a (potential) disease is diagnosed—even before the symptoms occur—the faster and more accurate the treatment can be. This in turn contributes to a prolonged life expectancy of the patient.
- Traditional forms of diagnostic instruments may be inaccurate. An inaccurate treatment assigned because of a wrong diagnosis can be very harmful to the patient. Therefore, using very sensitive and specific diagnostic instruments, an inaccurate treatment can be prevented before it causes serious damage [5, 6, 16].

The need for a more accurate, cost-saving, and fast diagnostic method is acute. Especially when we look on the market, type 2 diabetes mainly caused by overweight, less sport/movements, by smoking, the following customer pain and need can further be identified.

However, the identification and quantification of such diabetes-related biomarkers by using a novel very high sensitive polyfluorophor technologies was never done before and the aim of the present study is to describe the development of a novel detection conjugate offers the pre-eminent fluorescent labeling technology by using polyfluorophors.

## Improve speed and sensitivity of current in vitro diagnostic assays

Signal enhancement of in vitro diagnostic assay (IVD) is critical when the assay is operating at the limits of detection especially for the detection of low copy proteins of beta cell-related gene products usefully as screening markers for early onset of diabetes.

In the course of our study, we describe a novel detection conjugate technology by enhancing the light generated from existing diagnostic tests by up to 100–1000-fold; this novel detection conjugate will lower costs and boost performance and speed across the most widely used analysis platforms in the industry. This technology is versatile and agnostic technology that can be introduced into conventional tests, dramatically intensifying the resulting detection signals. In contrast to similar products for in vitro diagnosis, the novel detection conjugate by using polyfluorophores allows the detection of T2D-related biomarkers as already shown on prototype at an unprecedented level of speed and sensitivity in the femtogram level in different body fluids such as serum, plasma, saliva, etc. Thus, it significantly enables the earlier detection of T2D at a still pre-symptomatic stage and consequently increases healing success as well as life expectancy of patients. In addition, it is able to relief the burden of health insurers by lowering treatment costs. Finally, only a sample of body fluids from the patient will be required for testing with our product in contrast to more current time-consuming procedures such as OGTT for the investigations of the beta cell function.

## Description of the novel detection conjugates using the polyfluorophor technology

To improve the early diagnosis of T2D, the selection of predictive biomarkers must be carefully assessed and depends on different important parameters, e.g., sensitivity, specificity, positive and negative predictive value [7]. Unfortunately, biomarkers with ideal specificity and sensitivity are difficult to find. Sensitivity is the effectiveness of a test in detecting T2D in those who have the disease. Specificity is the extent to which a test gives negative results in those that are free of the disease. One potential solution is to use the combinatorial power of a large number of biomarkers, each of which alone may not offer satisfactory specificity and sensitivity to improve early diagnosis. Therefore, reliable and/or disease predictive multiplex assays with a very high sensitivity are urgently needed as common assays (ELISA, Western blot, etc.) allow only the quantification of a single analyte in nano- to picogram levels. Furthermore, they are inadequate to quantify low-abundance biomarker in body fluids like blood, plasma, urine, and saliva.

In the course of our research activities, we have developed a microchip technology in combination with a new light amplification technology by using polyfluorophors. Preliminary results showed that this prototype is able to significantly improve existing analytical multiplex assay systems in terms of sensitivity, enabling biomarker detection at femtogram level.

Antibody chip arrays represent an important new tool in validation of biomarker panels. This new techniques have a lot of benefits compare to the traditional common ELISA techniques [8–11]. In the frame of this article, a short review that focuses on the contributions of a novel protein chips array to the validation of a two novel diabetes type 2 biomarkers through antibody-based assays in the frame of a diabetes initiative was described. Of particular interest is the determination of the ration of active PPY content in human plasma samples from patient with T2D, T1D, and impaired glucose tolerance. The antigens and antibodies revealed by these studies are useful for clinical assay development, with enormous potential to aid in diagnosis, prognosis, disease staging, and treatment selection. The determination and characterization of this biomarker for beta cell function specifically addressed to disease type and stage are expected to enable personalized medicine, predictive diagnostics, and individualized curative therapies.

## Technical description of the novel polyfluorophor latex beat technology

**Fig. 2:**
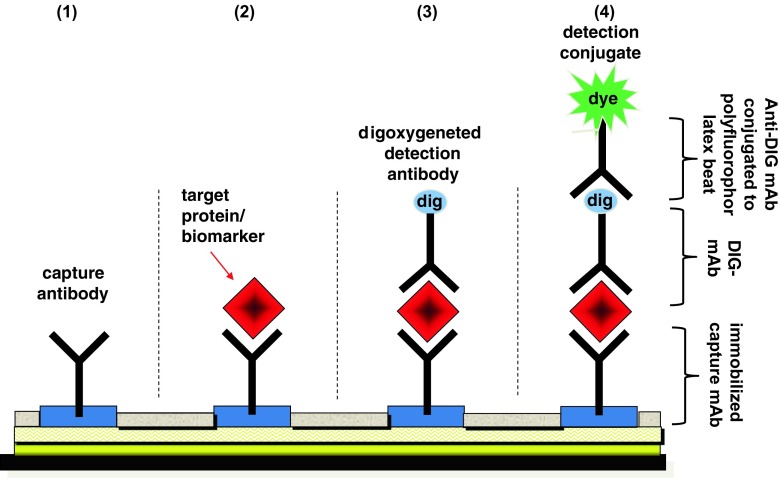
Schematic description of the novel polyfluorophor latex beat technology used for the quantification of pancreatic polypeptide-antigens in human plasma samples

In the frame of our project, we used an array of biotinylated antibodies, which have been immobilized through bonding to streptavidin-coated chip surface. In a second step, free spaces between the immobilized capture antibodies spot was blocked by coating with glucose and the chip was then finally completely dried. Chips under this condition are very stable. They can be stored for several years under RT and presents an excellent platform for further protein analyte profiling (Fig. 2).

The novel highly sensitive detection technique is based on an immune sandwich strategy. (1) In this system, a capture antibody is immobilized on a surface. (2) Then, a sample of body fluid is added and any present target biomarker within this sample binds to the capture antibody (first incubation). (3) In the next step, a detection antibody is introduced. This second antibody is labeled with a special anti-digoxin binding system, which will bind the target (second incubation). (4) Afterwards, a detection conjugate containing polyfluorescent dyes immobilized on latex beats is added, which is used to signal the presence of the target biomarker (third incubation). This signal is measured by a common scanner.

This approach has been used to great effect for the simultaneous detection of multiple PPY and GLP levels in biological samples. The highly specific antibody-complex recognition is ideal for detecting low-abundance PPY levels and holds much potential for clinical diagnostic application and discovery of therapeutic drug targets for prevention of beta cell functions in patients with T2D.

Limitations: Further development of the systems lies in the availability of high-quality Ab against the individual proteins [12–15]. At the present, it remains unfeasible to obtain more than few hundreds of different Ab that can recognize and capture various proteins with high affinity and specificity—factors that are essential for preventing cross reactivity. In the frame of our systems, two highly specific antibodies must be obtained for each captured protein. These must also recognized two different regions of the protein, each without masking the other binding domain.

### How can we achieve such a high sensitivity?

**Fig. 3:**
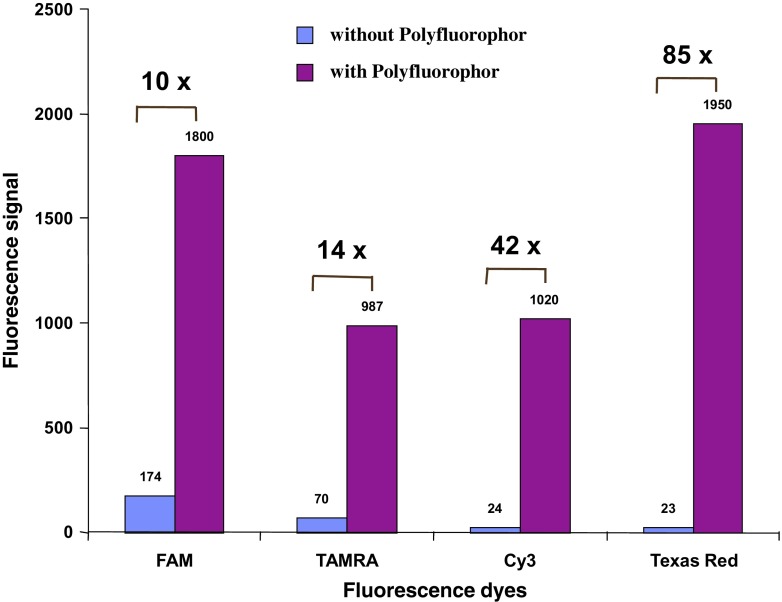
Fluorescence signal with and without polyfluorophor

Our first competitive advantage based on this strategy is the novel light amplification technology with a polyfluorophor-conjugated detection system. The optical enhancement of fluorescent dyes by polyfluorophor immobilized on a latex beat increases the detection signal up to 100 times (see Fig. 3). This “molecular antenna”—behavior of our polyfluorophor—forms the foundation of our technology and intellectual property base. However, future work will include the direct, covalent linkage of the polyfluorophor to the detection conjugate leading to increased signal amplification by the factor 1000.

Our second competitive advantage which increases the sensitivity further is the spotting technology. The immobilization of the capture antibody on a plate leads to a signal amplification by a factor of 100. Since the space the antibody is spotted on is much smaller than in conventional diagnostics instruments, it is possible to achieve equilibrium between antibody and target biomarker much faster. Therefore, the incubation period can be reduced from 3–4 h to 10 min.

Moreover, by using a special anti-digoxin binding system and highly specific detection antibodies, we can improve the signal-to-noise ratio and increase signal amplification by a factor of 1 million.

As a result, the cumulated signal amplification (higher sensitivity) can be calculated as follows: (100) 1000 × 100 × 1 m = 100 bn (10 bn)

**Fig. 4:**
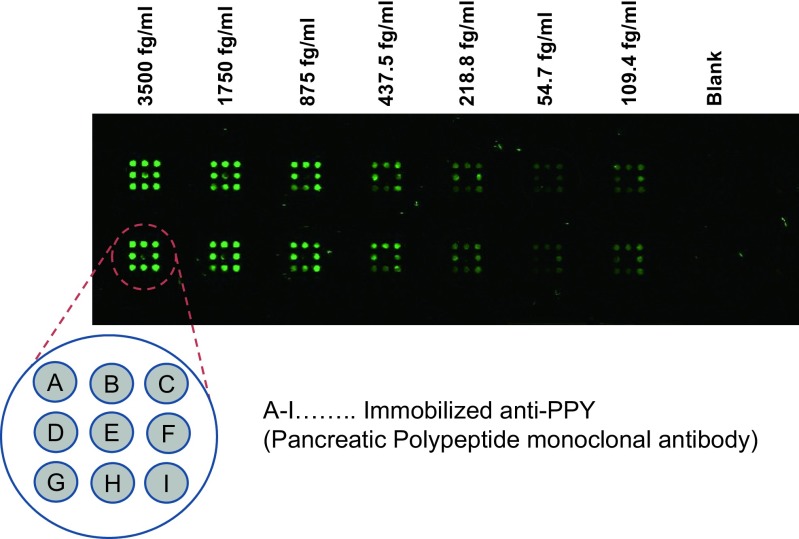
Quality characteristic of the calibration-curve of PPY used by the novel protein chip platform. Monoclonal capture Ab: Bio-anti-PPY, monoclonal detection Ab: DIG anti-PPY, Epitope: active PPY

Furthermore, as our new technology is not tied to a specific platform (e.g., MTP, glass plate for microscopy), it can be used to improve the detection signal of every common multiplex assay. However, as platform for our studies, we are currently using microtiter system (see Fig. 3). Each well within our multiplex protein chip is able to measure at the moment up to 9 data points simultaneously as shown in Fig. 4. Nevertheless, in the course of our research activities, we will choose an innovative multiplex assay platform which can be retooled for nearly any diagnostic biomarkers by replacing and optimizing the specific capture/detection conjugate as all other components will be universal (Table 1).

**Table 1:** Comparative results and characteristic between ELISA and the novel protein chip technology

	Sensitivity (%)	Specificity (%)	
ELISA	88	95	
Protein chip	92	95	
	Sample vol.	mAb vol.	Working time
ELISA	50–100 μl	0.25 μg/well	4–5 h
Protein chip	20 μl (1:1)	250 pg/dot	∼10 min

**Table:** 

Limit of detection:	10–50 fg/ml
Linear dynamic range:	0.05–1 pg/ml
Opt. spot conc.	50 μg/ml
Capture Ab:	Bio anti-PPY
Detection Ab:	DIG anti-PPY

## Discussion

**Fig. 5:**
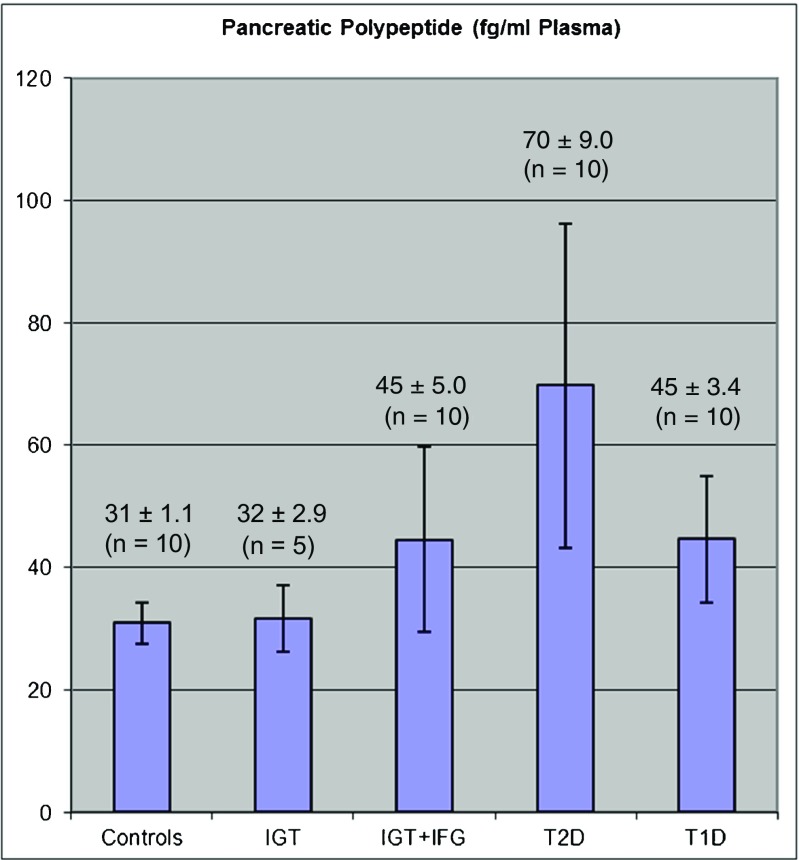
Validation of pancreatic polypeptide (*PPY*) in control patient, in patient with impaired glucose tolerance (*IGT*), impaired glucose tolerance + impaired fasten glucose (*IGT* + *IFG*), type 2 diabetes (*T2D*), and type 1 diabetes (*T1D*) by the novel micro chip assay

The detection of D2T in a pre-symptomatic stage leads to a significant advantage for patients: the earlier and more accurate the diagnosis the higher is the healing success. Moreover, the early detection of T2D with a greater accuracy permits earlier and more effective treatment and will consequently help to save treatment costs. This effect will be very attractive for health insurance companies as the burden of the health care system will be relieved. Furthermore, our new and innovative technologies will supplies diagnostic laboratories with a much sensitive and specific result compared to common diagnostic methods. Therefore, our study can make an accurate and fast diagnosis of T2D while cutting processing times and associated costs. At the first time, we select pancreatic polypeptide (PPY) which was already identified as a specific biomarker for beta cell dysfunction/beta cell failure (Fig. 5).

PPY was first isolated and sequenced 20 years ago, and in vitro and animal data have demonstrated that PPY has been a positive effect on the treatment of T2D. It increases beta cell mass by stimulating neogenesis and by inhibiting apoptosis of islets. It has been shown to have many effects:

1. PPY stimulates insulin secretion
2. Decreases glucagon levels
3. Reduces appetite
4. Able to reduce plasma glucose concentrations
5. It can stimulate beta cell neogenesis
6. May even be neuroprotective

It is no surprise that this peptide is of increasing interest as a target for the treatment of diabetes and the PPY system may provide a new therapeutic option for diabetes in the future. PPY are both rapidly degraded into inactive metabolites by the enzyme dipeptidyl peptidase-IV (DPP-IV). Incretin hormones are released in response to nutrient ingestion, which potentiate the glucose-induced insulin response. In humans, the incretin effect is mainly caused by two peptide hormones,

1. Glucose-dependent insulin-releasing polypeptide (GIP) which is secreted by K cells
2. Glucagon-like peptide-1 (PPY) which is mainly produced in the endocrine L cells.

Their effect is mediated through their binding with specific receptors, though part of their biological action may also involve neural modulation. GIP and in addition to its effects on insulin secretion, PPY exerts other significant actions, including stimulation of insulin biosynthesis, inhibition of glucagon secretion, inhibition of gastric emptying and acid secretion, reduction of food intake, and trophic effects on the pancreas. As the insulin tropic action of PPY is preserved in type 2 diabetic patients, this peptide was a candidate as a therapeutic agent for this disease. A number of pharmacological strategies have been developed to provide continuous delivery of PPY and to prevent degradation of PPY, including continuous administration of PPY, DPP-IV inhibitors, and DPP-IV-resistant PPY analogues. Recent results of the most clinically advanced incretin mimetic confirmed their efficacy to improve glycemic control in type 2 diabetic patients. Further results are expected to confirm the efficacy/safety profile of these compounds, and to find their place in the therapeutic strategy of type 2 diabetes.

The improvement of beta cell function can be indirectly observed from the increased insulin secretory capacity of humans receiving PPY or incretin mimetic that act like PPY. Furthermore, PPY inhibits glucagon secretion and rarely causes hypoglycemia. It may represent an attractive therapeutic method for type 2 diabetes because of its multiple effects, including a slowing of gastric emptying and the simulation of satiety by acting as a transmitter in the CNS. Native PPY is degraded rapidly upon intravenous or subcutaneous administration and is therefore not feasible for routine therapy. Long-acting PPY analogs (e.g., Liraglutide [Novo Nordisk, Copenhagen, Denmark]) and exenadin-4 (Exenatide [Eli Lilly, Indianapolis, IN]) that are resistant to degradation, called “incretin mimetics,” are being investigated in clinical trials. Dipeptidyl peptidase IV inhibitors (e.g., Vildagliptin [Novartis, Basel, Switzerland]) that inhibit the enzyme responsible for incretin degradation are also under study.
